# Differences in isolation rate and antimicrobial susceptibility of bacteria isolated from foals with sepsis at admission and after ≥48 hours of hospitalization

**DOI:** 10.1111/jvim.15692

**Published:** 2020-02-05

**Authors:** Mathijs J. P. Theelen, W. David Wilson, Barbara A. Byrne, Judy M. Edman, Philip H. Kass, Lapo Mughini‐Gras, K. Gary Magdesian

**Affiliations:** ^1^ Department of Equine Sciences Faculty of Veterinary Medicine, Utrecht University Utrecht The Netherlands; ^2^ Department of Infectious Diseases and Immunity Faculty of Veterinary Medicine, Utrecht University Utrecht The Netherlands; ^3^ Department of Medicine and Epidemiology School of Veterinary Medicine, University of California Davis California; ^4^ Department of Pathology, Microbiology & Immunology School of Veterinary Medicine, University of California Davis California; ^5^ Department of Population Health and Reproduction School of Veterinary Medicine, University of California Davis California; ^6^ Centre for Infectious Disease Control (CIb) National Institute for Public Health and the Environment (RIVM) Bilthoven The Netherlands

**Keywords:** amikacin, ampicillin, antimicrobial resistance, ceftiofur, chloramphenicol, enrofloxacin, gentamicin, healthcare‐associated infections, horse, hospital‐acquired infections, imipenem, neonate, nosocomial infections, penicillin, tetracycline, trimethoprim/sulfamethoxazole

## Abstract

**Background:**

Antimicrobial treatment protocols for foals with sepsis that do not improve clinically often are adjusted based on bacteriological and antimicrobial susceptibility testing results from samples collected at hospital admission.

**Objectives:**

To evaluate whether hospitalization for ≥48 hours affects bacteriological and antimicrobial susceptibility testing results.

**Animals:**

Two‐hundred sixty‐seven foals <30 days of age admitted to a neonatal intensive care unit and diagnosed with sepsis.

**Methods:**

Medical records were reviewed retrospectively to identify foals with sepsis and positive bacteriological cultures. Results from samples collected at hospital admission were compared to those collected ≥48 hours after admission. Logistic regression for clustered data and exact logistic regression were used for statistical analysis.

**Results:**

Three‐hundred fifty‐three unique bacterial isolates were obtained from 231 foals at hospital admission and 92 unique bacterial isolates were obtained from 57 foals after ≥48 hours of hospitalization. Relative isolation frequency after ≥48 hours of hospitalization increased for *Acinetobacter* spp., 0.6% versus 3.3% (odds ratio [OR], 7.63; 95% confidence interval [CI], 1.28‐45.45); *Enterococcus* spp., 4.8% versus 19.6% (OR, 5.37; 95% CI, 2.64‐10.90); *Klebsiella* spp., 5.1% versus 10.9% (OR, 2.27; 95% CI, 1.05‐4.89); *Pseudomonas* spp., 3.0% versus 7.6% (OR, 3.49; 95% CI, 3.49‐240.50); and *Serratia* spp., 3.0% versus 5.4% (OR, 20.23; 95% CI, 2.20‐186.14). Bacteria isolated after ≥48 hours of hospitalization were less susceptible to all tested antimicrobial drugs, except for imipenem.

**Conclusions and Clinical Importance:**

Decreased antimicrobial susceptibility of bacteria isolated after ≥48 hours of hospitalization provides a rationale for repeated bacteriological culture and susceptibility testing in hospitalized foals with sepsis.

**Abbreviations**
ARD: Antimicrobial removal device
CDC: Centers for Disease Control and Prevention
CI: confidence interval
CLSI: Clinical Laboratory Standards Institute
CRSE: cluster robust standard errors
HAI: health care‐associated infection
MIC: minimum inhibitory concentration
OR: odds ratio
TMS: trimethoprim/sulfamethoxazole
VMTH: Veterinary Medical Teaching Hospital
WHO: World Health Organization

## 1 INTRODUCTION

Up to 60% of foals admitted to an intensive care unit in Florida were considered septic at hospital admission.^1^ *Escherichia coli* is the bacterium most commonly isolated from foals with sepsis in most studies.^2^, ^3^, ^4^, ^5^, ^6^, ^7^, ^8^ Antimicrobial susceptibility of bacteria isolated from foals with sepsis varies among different geographic regions.^3^, ^5^, ^6^, ^8^, ^9^, ^10^, ^11^, ^12^ Temporal trends toward increased antimicrobial resistance to frequently used antimicrobial drugs, such as gentamicin, amikacin, and ceftiofur, have been identified.^12^ This finding highlights the need to perform bacteriological culture and susceptibility testing in foals suspected of sepsis.

Bacteriological and antimicrobial susceptibility testing is performed routinely on samples collected from foals with suspected sepsis at hospital admission.^13^ While awaiting test results, the choice of antimicrobials to initiate treatment typically is based on historical data on antimicrobial susceptibility patterns of pathogens causing sepsis of foals in that geographic location.^3^, ^5^, ^6^, ^8^, ^9^, ^10^, ^11^, ^12^ The antimicrobial treatment regimen then is adjusted as necessary based on the results of culture and susceptibility testing of admission samples. Although this approach results in a successful outcome in 65% of affected foals, 35% fail to show clinical improvement despite treatment with antimicrobials that should be effective based on susceptibility testing of bacteria isolated from admission samples.^13^ Clinicians then may opt to give the chosen antimicrobial protocol more time to be effective or adjust the treatment protocol to include other antimicrobials to which the bacteria isolated from admission samples were susceptible. Both of these approaches assume that the bacterial species infecting the foal and the antimicrobial susceptibility profile of these bacteria remain the same as those obtained from admission samples.

In adult horses, hospitalization and treatment with antimicrobial drugs create selection pressure on bacteria, leading to the development of antimicrobial resistance.^14^, ^15^, ^16^, ^17^ Several studies have reported on isolation rate and susceptibility patterns of bacteria isolated from foals with sepsis.^2^, ^3^, ^5^, ^6^, ^8^, ^9^, ^10^, ^11^, ^12^ However, the effect of hospitalization and antimicrobial treatment before the time of sampling on culture results has not been investigated in foals with sepsis.

The Centers for Disease Control and Prevention (CDC) defines health care‐associated infections (HAIs) as localized or systemic conditions resulting from an adverse reaction to the presence of an infectious agent(s) or its toxins in which case there is no evidence that the infection was present or incubating at the time of hospital admission.^18^ Critically ill‐human patients admitted to intensive care units are at risk of developing HAIs, frequently related to particular surgical and medical procedures, which often involve specific species or strains of bacteria that are resistant to many antimicrobial drugs.^19^ The same is likely true for foals admitted to neonatal intensive care facilities, but data to support this assumption currently are lacking. The main purpose of our study was to compare isolation rates and antimicrobial susceptibility patterns of bacteria isolated from foals with sepsis between samples collected on hospital admission and after ≥48 hours of hospitalization. The 2nd aim was to determine if HAIs occurred in foals after ≥48 hours of hospitalization.

The hypotheses were that different bacterial species would be isolated after ≥48 hours of hospitalization compared to samples collected at hospital admission and that these bacteria would be more resistant to antimicrobials. Also, we hypothesized that a large proportion of positive bacterial cultures after ≥48 hours of hospitalization potentially would be the result of HAIs.

## 2 MATERIALS AND METHODS

### 2.1 Study design and case selection

A retrospective review of medical records of foals ≤30 days of age admitted to the William R. Pritchard Veterinary Medical Teaching Hospital (VMTH), University of California (Davis, California), between January 1, 1990 and December 31, 2015 was performed. Data recorded in the medical records at admission and during hospitalization of the foal were retrieved from the hospital veterinary medical information system. Records for those foals with a clinical diagnosis of sepsis, confirmed by a positive bacteriological culture from blood or normally sterile internal sites (abdominal fluid, pleural fluid, cerebrospinal fluid, IV catheter tips, and joints) before 30 days of age, were selected for further evaluation. For each case, data on year of hospital admission, age, body temperature, results from blood tests (eg, hematology, serum fibrinogen and glucose concentrations, blood pH, pCO_2_, and bicarbonate concentration), presence or absence of scleral injection, petechial hemorrhage, anterior uveitis, diarrhea, respiratory distress, neurologic signs or joint swelling, information on initial antimicrobial treatment and results from all bacteriological cultures, and susceptibility testing performed during hospitalization (including culture site and time of sampling relative to hospital admission) were collected. Results from culture of samples collected at necropsy also were included. To minimize the likelihood of including contaminated samples, results only were included if isolates were identified from >1 normally sterile site (ie, liver, spleen, kidney, lungs, heart, meninges, body cavity, or joints). Carcasses of foals were kept refrigerated after death or euthanasia until the necropsy was performed on the day of death or the next day. Cases were included only if foals showed ≥5 clinical or pathologic signs of systemic sepsis at the time of sample collection, such as fever (>38.9°C), neutropenia, or neutrophilia (<4000 or > 12 000 neutrophils/μL), increased band neutrophil count (>50 band neutrophils/μL), presence of toxic changes in neutrophils, hyperfibrinogenemia (>400 mg/dL), hypoglycemia (<80 mg/dL), metabolic acidosis, scleral injection, petechial hemorrhage, anterior uveitis, diarrhea, respiratory distress, neurologic signs (hypotonia, lethargy, coma, or seizures), or joint swelling. To address the main goal of the study, all samples collected on the day of hospital admission were included in the group of “samples collected at hospital admission.” All samples collected after ≥48 hours of hospitalization were included in the group of “samples collected after ≥48 hours of hospitalization.” Samples collected after the day of hospital admission but before 48 hours of hospitalization were excluded from the study to prevent overlap. To address the 2nd aim of the study, samples were included only if they were collected after ≥48 hours of hospitalization and, from the same foals, bacteriological cultures also had been performed at hospital admission.

### 2.2 Bacterial isolation, identification, classification, and antimicrobial susceptibility testing

Bacterial isolation, identification, and classification were performed as described previously.^12^ The broth microdilution Sensititre procedure (ThermoFisher Scientific, Cleveland, Ohio) was used for antimicrobial susceptibility testing, following Clinical Laboratory Standards Institute (CLSI) protocols.^20^ The minimum inhibitory concentration (MIC) was recorded as the lowest concentration of antimicrobial drug that inhibited visible growth of bacteria or 80% inhibition in the case of trimethoprim/sulfamethoxazole (TMS). Breakpoints published in the 3rd edition of the “Performance Standards for Antimicrobial Disk and Dilution Susceptibility Tests for Bacteria Isolated From Animals” by CLSI were used to determine susceptibility for all isolates included in the study, occasionally modified based on research in horses (see Table S1).^20^ For foals that were treated with combinations of antimicrobial drugs, an isolate was considered to be susceptible to this combination of drugs if its MIC for at least 1 of the drugs in the combination was equal to or less than the breakpoint.

### 2.3 Antimicrobial drugs

The following antimicrobial drugs were evaluated for activity against bacteria isolated from foals with sepsis: amikacin, ampicillin, ceftiofur, chloramphenicol, enrofloxacin, gentamicin, imipenem, penicillin, tetracycline, and TMS. Based on the measured antimicrobial activity of these drugs, the susceptibility of individual isolates to the following combinations of drugs, which frequently are used to treat foals with sepsis, was predicted: amikacin + penicillin, amikacin + ampicillin, amikacin + ceftiofur, gentamicin + penicillin, and gentamicin + ampicillin.

### 2.4 Positive bacterial cultures after ≥48 hours of hospitalization

Positive bacterial culture results after ≥48 hours of hospitalization were compared to results from samples collected from the same foals at hospital admission. All isolates were classified as belonging to 1 of the 4 categories. The 1st category included isolates that were obtained only from samples collected after ≥48 hours of hospitalization. Samples collected on the day of hospital admission from the same foals were either culture‐negative or were positive for other organisms that were no longer isolated after ≥48 hours of hospitalization. The 2nd category included isolates that were obtained from samples collected at both time points and on both occasions were susceptible to the initially administered antimicrobials. The 3rd category included bacteria that also were isolated on both time points, but these isolates were susceptible to the administered antimicrobials on hospital admission and resistant after ≥48 hours of hospitalization. The 4th category included isolates that also were obtained at both time points, but were, on both occasions, resistant to the initial antimicrobial treatment.

### 2.5 Statistical analysis

Logistic regression using cluster robust SE estimation was used to assess the association between isolation frequency and antimicrobial susceptibility of bacteria isolated at hospital admission as compared to those isolated after ≥48 hours of hospitalization (Stata/IC 14.1).^21^, ^22^ Several potential confounders of these differences were identified before analysis of the data: “antemortem versus postmortem culture,” “culture site,” “year of culture,” and for the detection of differences in susceptibility patterns, “bacterial species isolated” also were identified. The potential confounding effects of these variables were assessed by comparing the results of the single‐variable analysis to those of the multivariable analysis, including the potential confounder as covariate. A change of ≥10% in the odds ratio (OR) was considered evidence of sufficient confounding to justify retention of the variable in the model regardless of its statistical significance; otherwise, the variable was excluded from the model. Because a strong association was found between time of sampling (“hospital admission” versus “after ≥48 hours of hospitalization”) and the variables “antemortem versus postmortem culture” and “culture site” (eg, postmortem cultures were overrepresented in the group of “samples collected after ≥48 hours of hospitalization” and blood cultures were overrepresented in the group of “samples collected at hospital admission”), the method described above could not be applied to “antemortem versus postmortem culture” and “culture site.” Therefore, a chi‐square test was used to assess the association of these variables with isolation frequency and antimicrobial susceptibility within the group of “samples collected at hospital admission” only. Because they were not found to be significantly associated with either isolation frequency or antimicrobial susceptibility, both variables were excluded from further analyses.

Inclusion or exclusion of the covariates is shown in Tables 1, 2, 3. When some of the cells formed by the outcome and predictor variable had no observations, exact logistic regression was used instead of ordinary logistic regression; correction for within‐cluster correlation was maintained. The statistical methods used are noted in the tables presenting the results (see Tables 1, 2, 3). Associations are expressed as ORs with 95% confidence intervals (CIs). Statistical significance was defined as *P* < .05.

**Table 1:** Isolation frequency of bacteria cultured from foals with sepsis at admission versus after >48 hours of hospitalization

	Admission (n = 353)		>48 hours of hospitalization (n = 92)			
Bacterial species	Number of isolates	Percentage of total isolates (%)	Number of isolates	Percentage of total isolates (%)	Odds ratio	95% confidence interval
Gram‐negative bacteria	266	75.4	65	70.7	1.25^a^	0.74‐2.11
*Escherichia coli*	130	36.7	30	32.6	0.83^a^	0.51‐1.36
*Klebsiella* spp.	18	5.1	10	10.9	2.27^a^	1.05‐4.89
*Enterobacter* spp.	9	2.5	6	6.5	2.67^a^	0.91‐7.83
*Salmonella* spp.	6	1.7	0	0	3.00^b^	0.00‐57.00
*Pantoea* spp.	4	1.1	1	1.1	0.86^a^^,^ c	0.09‐8.06
*Proteus* spp.	4	1.1	2	2.2	1.94^a^	0.33‐11.51
*Serratia* spp.	1	0.3	5	5.4	20.23^a^	2.20‐186.14
*Actinobacillus* spp.	69	19.5	0	0	0.15^b^	0.00‐0.91
*Aeromonas* spp.	6	1.7	0	0	0.33^b^	0.00‐6.33
*Pasteurella* spp.	5	1.4	0	0	0.57^d^	0.00‐3.15
*Acinetobacter* spp.	2	0.6	3	3.3	7.63^a^	1.28‐45.45
*Pseudomonas* spp.	1	0.3	7	7.6	3.49^a^	3.49‐240.50
Other Gram‐negative isolates	11	3.1	1	1.1	‐	‐
Gram‐positive bacteria	87	24.6	27	29.3	1.25^a^	0.74‐2.11
*Streptococcus* spp.	41	11.6	4	4.3	0.35^a^	0.13‐0.91
*Enterococcus* spp.	17	4.8	18	19.6	5.37^a^^,^ c	2.64‐10.90
*Staphylococcus* spp.	19	5.4	5	5.4	1.01^a^	0.31‐3.26
*Bacillus* spp.	7	2.0	0	0	0.39^c^	0.00‐2.04
Other Gram‐positive isolates	3	0.8	0	0	‐	‐

a Cluster robust SE (CRSE).

b Exact logistic regression (including correction for clustering) (CRSE not possible because of no observations in one of the groups).

c The variable “year of culture” was included in the model as a covariable.

d Exact logistic regression (ignoring clustering, because of sparse data the clustering for those variables could not be evaluated).

**Table 2:** Susceptibility of bacteria cultured from foals with sepsis at admission versus after >48 hours of hospitalization

Antimicrobial drug (combinations)	Admission		>48 hours of hospitalization			
	Total number of isolates	Number of susceptible isolates	Total number of isolates	Number of susceptible isolates	Odds ratio	95% confidence interval
Amikacin	334	229 (68.6%)	85	36 (42.4%)	0.11^a^^,^ b	0.04‐0.27
Ampicillin	331	229 (69.2%)	84	24 (28.6%)	0.31^a^^,^ b	0.16‐0.58
Ceftiofur	331	301 (90.9%)	85	42 (49.4%)	0.03^a^^,^ b ^,^ c	0.01‐0.11
Chloramphenicol	326	277 (85.0%)	83	37 (44.6%)	0.22^a^^,^ b	0.12‐0.41
Enrofloxacin	330	289 (87.6%)	84	56 (66.7%)	0.28^a^	0.16‐0.51
Gentamicin	334	229 (68.6%)	85	26 (30.6%)	0.17^a^^,^ b	0.08‐0.34
Imipenem	289	272 (94.1%)	76	59 (77.6%)	0.49^a^^,^ b	0.18–1.33
Penicillin	324	128 (39.5%)	76	10 (13.2%)	0.23^a^	0.11‐0.52
Tetracycline	306	237 (77.5%)	81	27 (33.3%)	0.26^a^^,^ b	0.14‐0.50
Trimethoprim/sulfamethoxazole	334	215 (64.4%)	85	19 (22.4%)	0.22^a^	0.11‐0.45
Amikacin + penicillin	331	301 (90.9%)	77	45 (58.4%)	0.15^a^^,^ b	0.07‐0.32
Amikacin + ampicillin	334	313 (93.7%)	84	48 (57.1%)	0.13^a^^,^ b	0.06‐0.29
Amikacin + ceftiofur	333	312 (93.7%)	85	53 (62.4%)	0.04^a^^,^ b	0.01‐0.18
Gentamicin + penicillin	329	285 (86.6%)	77	35 (45.5%)	0.18^a^^,^ b	0.09‐0.35
Gentamicin + ampicillin	333	290 (87.1%)	84	37 (44.0%)	0.19^a^^,^ b	0.10‐0.36

a Cluster robust SE.

b “Bacterial species isolated” was included in the model for the statistical analysis as a covariable.

c The variable “year of culture” was included in the model for the statistical analysis as a covariable.

**Table 3:** Susceptibility of *E. coli* cultured from foals with sepsis at admission vs. after >48 hours of hospitalization

Antimicrobial drug (combinations)	Admission		>48 hours of hospitalization			
	Total number of *E. coli*	Number of susceptible *E. coli*	Total number of *E. coli*	Number of susceptible *E. coli*	Odds ratio	95% confidence interval
Amikacin	124	114 (91.9%)	29	17 (58.6%)	0.12^a^	0.04‐0.35
Ampicillin	124	87 (70.2%)	29	12 (41.4%)	0.30^a^	0.13‐0.71
Ceftiofur	124	123 (99.2%)	29	23 (79.3%)	0.02^a^^,^ b	<0.01‐0.39
Chloramphenicol	121	95 (78.5%)	29	11 (37.9%)	0.17^a^	0.07‐0.38
Enrofloxacin	124	121 (97.6%)	29	27 (93.1%)	0.26^a^^,^ b	0.03–1.98
Gentamicin	124	108 (87.1%)	29	12 (41.4%)	0.10^a^	0.04‐0.25
Imipenem	113	109 (96.5%)	27	27 (100%)	1.28^c^	0.21 to ∞
Penicillin^d^	124	0 (0%)	27	0 (0%)	NA	NA
Tetracycline	115	84 (73.0%)	27	9 (33.3%)	0.18^a^	0.08‐0.45
Trimethoprim/sulfamethoxazole	124	86 (69.4%)	29	10 (34.5%)	0.23^a^	0.10‐0.52
Amikacin + penicillin	124	114 (91.9%)	28	17 (60.7%)	0.14^a^	0.05‐0.38
Amikacin + ampicillin	124	119 (96.0%)	29	19 (65.5%)	0.08^a^	0.02‐0.28
Amikacin + ceftiofur	124	123 (99.2%)	29	26 (89.7%)	0.07^a^	0.01‐0.68
Gentamicin + penicillin	124	108 (87.1%)	28	12 (42.9%)	0.11^a^	0.05‐0.26
Gentamicin + ampicillin	124	109 (87.9%)	29	13 (44.8%)	0.11^a^	0.05‐0.27

a Cluster robust SE.

b The variable “year of culture” was included in the model as a covariable.

c Exact logistic regression (ignoring clustering, because of sparse data the clustering for those variables could not be evaluated).

d *E. coli* was always classified as resistant to penicillin (regardless of MIC value).

## 3 RESULTS

A total of 445 bacterial isolates from 267 foals were included in this study. Three‐hundred fifty‐three isolates were obtained from samples collected from 231 foals (median age, 4 days; range, 0‐28 days) on the day of hospital admission and therefore were included in the group of “samples collected at hospital admission.” Of the isolates included in this group, 286 were obtained from samples collected antemortem and 67 were obtained from samples collected postmortem. The majority of these bacteria were isolated from blood cultures (n = 231), but bacteria also were isolated from various organs at necropsy (n = 67), joint aspirates (n = 30), peritoneal fluid samples (n = 15), IV catheter tips (n = 4), pleural fluid samples (n = 3), and cerebrospinal fluid samples (n = 3). Ninety‐two isolates were obtained from samples collected from 57 foals (median age, 6 days; range, 2‐29 days) after ≥48 hours of hospitalization (median time of sampling postadmission, 5 days; range, 3‐30 days) and therefore were included in the group “samples collected after ≥48 hours of hospitalization.” Of the isolates included in this group, 46 were obtained from samples collected antemortem and 46 were obtained from samples collected postmortem. The majority of these bacteria were isolated from various organs at necropsy (n = 46), but bacteria also were isolated from blood cultures (n = 11), joint aspirates (n = 11), peritoneal fluid samples (n = 11), and IV catheter tips (n = 13). Of 57 foals with positive cultures after ≥48 hours of hospitalization, 21 had positive cultures at both hospital admission and after ≥48 hours of hospitalization and therefore were included in both groups, 30 had negative cultures at admission, and 6 had no cultures performed at admission.

### 3.1 Isolation frequency

*Escherichia coli* was isolated most frequently from samples collected on the day of hospital admission, followed by *Actinobacillus* spp. and *Streptococcus* spp. After ≥48 hours of hospitalization, *E. coli* remained the most frequently isolated bacterium, followed by *Enterococcus* spp. and *Klebsiella* spp.

The odds of *Actinobacillus* spp. (OR, 0.15; 95% CI, 0.00‐0.91) and *Streptococcus* spp. (OR, 0.35; 95% CI, 0.13‐0.91) being isolated from samples collected after ≥48 hours of hospitalization significantly decreased (Table 1). The odds of *Acinetobacter* spp. (OR, 7.63; 95% CI, 1.28‐45.45), *Enterococcus* spp. (OR, 5.37; 95% CI, 2.64‐10.90), *Klebsiella* spp. (OR, 2.27; 95% CI, 1.05‐4.89), *Pseudomonas* spp. (OR, 3.49; 95% CI, 3.49‐240.50), and *Serratia* spp. (OR, 20.23; 95% CI, 2.20‐186.14) being isolated from samples after ≥48 hours of hospitalization all significantly increased (Table 1).

### 3.2 Antimicrobial susceptibility

Susceptibility data, specified by antimicrobial drug or combination of antimicrobial drugs, are presented in Table 2. The following antimicrobial drugs and their combinations were predicted to have an efficacy of >90% against bacteria isolated at hospital admission: amikacin + ampicillin (93.7%), amikacin + ceftiofur (93.7%), amikacin + penicillin (90.9%), ceftiofur (90.9%), and imipenem (94.1%). None of the antimicrobial drugs or their combinations had a predicted efficacy of >90% against bacteria isolated from samples collected after ≥48 hours of hospitalization. The odds of bacteria isolated after ≥48 hours of hospitalization being susceptible to individual antimicrobial drugs or combinations of drugs tested in the study decreased significantly compared to bacteria isolated at hospital admission for all drugs and drug combinations (range of ORs, 0.03 to 0.31), except for imipenem (OR, 0.49; 95% CI, 0.18‐1.33).

*Escherichia coli* was the only bacterial species for which the number of isolates was high enough to make a meaningful comparison between hospital admission and after ≥48 hours of hospitalization. Thus, we also have included separate susceptibility data for *E. coli* (Table 3). The odds that *E. coli* isolated after ≥48 hours of hospitalization were susceptible to individual antimicrobial drugs or combinations of drugs also decreased significantly compared to *E. coli* isolated at hospital admission for all drugs and drug combinations (range of ORs, 0.02‐0.30), except for enrofloxacin (OR, 0.26; 95% CI, 0.03‐1.98) and imipenem (OR, 1.28; 95% CI, 0.21 to ∞).

### 3.3 Positive bacterial cultures after ≥48 hours of hospitalization

Fifty‐one foals had positive cultures after ≥48 hours of hospitalization and also had cultures performed at hospital admission, resulting in 82 isolates that were included in this part of the study. Of these 51 foals, 21 foals had positive cultures both at hospital admission and after ≥48 hours of hospitalization. Thirty foals had negative cultures at admission, but positive cultures after ≥48 hours of hospitalization.

For the 82 bacteria isolated after ≥48 hours of hospitalization, we compared the results of bacteriological culture and susceptibility testing after ≥48 hours of hospitalization to the results of samples collected from the same foals at hospital admission.

Seventy (85.3%) of 82 isolates were found only in samples collected after ≥48 hours of hospitalization. Samples collected on the day of hospital admission from the same foals were either culture‐negative or positive for other organisms that were no longer isolated after ≥48 hours of hospitalization. Five (6.1%) of 82 isolates were obtained at both time points and on both occasions were susceptible to the antimicrobials initially administered. Four (4.9%) of 82 isolates also were obtained at both time points, but were susceptible to the antimicrobials initially administered on hospital admission and resistant after ≥48 hours of hospitalization. Three (3.7%) of 82 isolates also were obtained at both time points, but were resistant to the initial antimicrobial treatment on both occasions.

## 4 DISCUSSION

Several studies have reported on isolation frequency and susceptibility of bacteria obtained from foals with sepsis.^2^, ^3^, ^5^, ^6^, ^8^, ^9^, ^10^, ^11^, ^12^ However, none of these studies has examined the association of time of sampling during hospitalization on the results. In adult horses, *E coli* bacteria isolated from fecal samples were more resistant after a period of hospitalization and treatment with antimicrobials, demonstrating the effect on bacteriological culture and susceptibility testing results also seen in our study of foals with sepsis.^14^, ^15^, ^16^, ^17^

The odds on nonsurvival were 2.26 times higher in bacteremic foals compared to foals with negative blood cultures in a study performed in Florida, emphasizing the necessity of empirical selection of antimicrobial drugs to initiate treatment in foals with sepsis.^1^ This initial antimicrobial treatment regime should be reviewed frequently and adjusted as necessary, particularly in cases that fail to improve clinically, because the likelihood of survival for foals with sepsis treated with antimicrobials for which all infecting bacteria are susceptible is 65% compared to 42% in foals for which at least 1 of the infecting bacteria is resistant to initial treatment.^13^ It is therefore important to know which bacteria are most likely to be involved and what their expected susceptibility patterns are at different points in time during hospitalization. Our study showed that isolation frequency and antimicrobial susceptibility of bacteria differed significantly between samples collected at hospital admission and after ≥48 hours of hospitalization. This finding indicates that selection of antimicrobial drugs to treat foals with on‐going sepsis during hospitalization cannot be based solely on culture results from samples collected at the time of hospital admission.

Of the drugs included in our study, ceftiofur and enrofloxacin are classified as “highest priority critically important antimicrobials” by the World Health Organization (WHO), which means they are regarded as critically important to human health.^23^ Amikacin, ampicillin, gentamicin, and imipenem are classified as “high priority critically important antimicrobials”. Chloramphenicol, penicillin, tetracycline, and TMS are “highly important antimicrobials” according to the WHO. The use of “highest priority critically important antimicrobials” in horses should be restricted, according to the WHO, and should be reserved only for cases for which no alternative antimicrobials are effective, and only after appropriate susceptibility testing. In our opinion, this policy also should be applied to imipenem, because it is the only “high priority critically important antimicrobial” we tested that is listed as the sole treatment available for specific diseases in humans.

### 4.1 Isolation frequency

After ≥48 hours of hospitalization, the odds of samples being positive for *Actinobacillus* spp. and *Streptococcus* spp. significantly decreased compared to samples collected at admission. These bacterial species typically are susceptible to antimicrobial drugs commonly used for initial treatment of foals with sepsis, such as the combination of amikacin and ampicillin.^12^ Therefore, it is not surprising that in samples collected after ≥48 hours of hospitalization these bacteria were isolated less frequently.

After ≥48 hours of hospitalization, the odds of bacterial cultures being positive for *Acinetobacter* spp., *Enterococcus* spp., *Klebsiella* spp., *Pseudomonas* spp., and *Serratia* spp. all significantly increased. A high proportion of these species of bacteria are known to show intrinsic or acquired resistance to many antimicrobial drugs, including those commonly used in initial treatment protocols for foals with sepsis.^24^, ^25^, ^26^, ^27^, ^28^, ^29^ These bacterial species were responsible for a large proportion of the HAIs in a large study in humans: *Enterococcus* spp., 13.9%; *Klebsiella* spp., 8%; *Pseudomonas* spp., 7.5%; *Serratia* spp., 2.1%; and *Acinetobacter* spp., 1.8%.^30^ In equine medicine, there also are several reports on the role of these bacteria in HAIs. A study on *Acinetobacter baumanni* isolates from companion animals and horses in Switzerland found that a majority of these infections were hospital‐acquired.^31^ In a study of surgical site infection after laparotomy in horses, *Enterococcus* spp. were the 2nd most commonly isolated bacteria.^32^ *Klebsiella* spp. were identified as causative organisms of pneumonia in 11 horses that had undergone mechanical ventilation under general anesthesia.^33^ The relatively high proportion of bacterial species that are known to frequently cause HAIs isolated in our study from samples after ≥48 hours of hospitalization suggests that HAIs could also play an important role in equine neonatal care. Further genotypic characterization would be required to confirm this hypothesis.

### 4.2 Antimicrobial susceptibility

Bacteria isolated after ≥48 hours of hospitalization were less susceptible to all antimicrobial drugs and combinations evaluated in our study compared to those isolated at admission. This decreased susceptibility was significant for all drugs and drug combinations, except for imipenem. None of the antimicrobial drugs or their combinations were predicted to have an efficacy of >90% against bacteria isolated after ≥48 hours of hospitalization. Susceptibility patterns of these bacteria were unpredictable. This observation can be explained in part by the different bacterial species that were isolated. Antimicrobial treatment was initiated in all foals included in our study at hospital admission after collection of the 1st sample for bacteriological culture and susceptibility testing. This approach likely led to an antimicrobial selection pressure, favoring growth of resistant bacterial populations, as is also seen in studies in adult horses.^14^, ^15^, ^16^, ^17^ Therefore, the bacterial species and strains isolated after ≥48 hours of hospitalization frequently were more resistant to multiple antimicrobial drugs compared to the species and strains isolated at hospital admission. However, the results for *E. coli* clearly indicate that, even within the same bacterial species, the odds of being susceptible significantly decreased between admission and ≥48 hours after admission (Table 3), indicating selection of more resistant strains, development of acquired resistance, or both.

### 4.3 Positive bacterial cultures after ≥48 hours of hospitalization

From the time of hospital admission, all foals included in our study were treated with antimicrobial drugs. Therefore, in all cases in which samples were collected during hospitalization, the foals had been treated with antimicrobials before collection of these samples. Negative cultures resulted after >48 hours of hospitalization in most foals. However, in 57 foals, samples submitted for bacteriological culture and antimicrobial susceptibility testing after ≥48 hours of hospitalization were positive (n = 92 isolates).

The 2nd aim of our study was to determine If HAIs occurred in foals after ≥48 hours of hospitalization by comparing results from cultures after ≥48 hours of hospitalization to test results from samples collected from the same foals at hospital admission. In 6 foals, no bacteriological culture was performed at hospital admission. Bacteria isolated from these foals (n = 10 isolates) therefore were excluded from this part of the study. The remaining 82 bacteria that were isolated after ≥48 hours of hospitalization were divided into 4 categories.

The 1st category included bacteria that were isolated only from samples collected after ≥48 hours of hospitalization. Samples collected on the day of hospital admission from the same foals were either culture‐negative or were positive for other organisms that were eliminated by the initial antimicrobial treatment and were therefore no longer isolated after ≥48 hours of hospitalization. Two possible explanations exist for inclusion in this category. The 1st explanation is that in these cases the sample collected at hospital admission was a false negative and the infection was not cleared by the initial antimicrobial treatment. The 2nd explanation is that these foals acquired an infection during hospitalization (HAIs). Given the study design, it is impossible to distinguish between these 2 possible explanations. Seventy of 82 isolates belong to this category (85.4%).

The 2nd category of positive samples after ≥48 hours of hospitalization includes presumed treatment failures (n = 5/82; 6.1%). The same bacteria were isolated from samples collected at both time points and were, on both occasions, susceptible to the administered antimicrobials.

The 3rd category includes bacteria that were isolated at both time points and were susceptible to the administered antimicrobials on hospital admission, but resistant after ≥48 hours of hospitalization. These bacteria potentially acquired resistance during hospitalization and antimicrobial treatment (n = 4/82; 4.9%), but further genotypic characterization would be required to confirm this possibility. Two other explanations for inclusion in this category are possible. First, it is possible that >1 morphologically identical strain of a particular bacterial species (and therefore >1 susceptibility pattern) was present in both samples, but only 1 of these strains was selected for susceptibility testing. Selection of different colonies then could have led to different susceptibility results. And 2nd, foals could have become infected with a more resistant strain of the same bacterial species during hospitalization (HAI) resulting in a different antimicrobial susceptibility pattern.

The 4th category of positive samples collected ≥48 hours of hospitalization includes bacteria that were not eliminated because they were resistant to the initial antimicrobial treatment on hospital admission (n = 3/82; 3.7%).

Given the study design, and by using the CDC definition of HAIs,^18^ it is impossible to determine with 100% certainty if a positive culture after ≥48 hours of hospitalization included in category 1 is the result of an HAI. However, the numbers of positive cultures after ≥48 hours of hospitalization included in category 2 (n = 5) and 4 (n = 3) were very low, suggesting it is rare for susceptible bacteria not to be eliminated by the initial antimicrobial treatment and it is equally rare for bacteria to be resistant to initial antimicrobial treatment. One of these scenarios would need to be the case for isolates that were included in category 1 as a result of a false negative culture at hospital admission. Therefore, we conclude that the majority of samples included in category 1 are most likely the result of HAIs. However, genotypic characterization would be required to confirm this hypothesis with certainty.

These findings support the conclusion that HAIs with resistant strains of bacteria potentially play an important role in equine neonatal medicine.

### 4.4 Limitations

We acknowledge that several aspects of the design of our study could have influenced the results obtained. First, only isolates originating from samples from foals treated at the University of California‐Davis VMTH were included, which could have led to geographically restricted findings. Second, our study was largely based on a retrospective review of medical records, and therefore cases for which essential information was missing were excluded. Given the retrospective nature of our study, we did not have follow‐up samples for bacteriological culture and susceptibility testing available for all foals included in the study. At hospital admission, blood cultures were collected routinely in foals suspected of sepsis, whereas later sampling during hospitalization was based on the clinical situation (eg, poor treatment response). This situation potentially could have created substantial sampling bias and prevented conclusions being drawn regarding potential mechanisms for the observed differences in isolation frequency and antimicrobial susceptibility. However, this factor does not restrict the clinical value of the data in guiding clinicians who need to decide whether to adjust antimicrobial treatment protocols in foals with on‐going sepsis. Not all susceptibility testing was performed at the same time, although the same methods were used throughout the study and the same interpretation criteria regarding antimicrobial susceptibility were applied to all isolates included in the study.^20^ Administration of antimicrobial drugs before hospitalization could have influenced susceptibility profiles of bacteria isolated at hospital admission. Data on antimicrobial treatment before admission were not available for all cases and could not be taken into account. Antimicrobial removal devices (ARD) were not consistently used for blood cultures throughout the study period. Without use of an ARD, inhibition of growth of susceptible bacteria in vitro may have occurred and given false negative culture results in some cases. Samples collected postmortem may have a higher risk of contamination compared to samples collected antemortem. To minimize the risk of this factor influencing our results, isolates collected postmortem only were included if they were isolated from at least 2 normally sterile sites.

### 4.5 Conclusions and clinical relevance

Susceptibility patterns of bacteria isolated after ≥48 hours of hospitalization were less predictable than those of foals tested at the time of admission, and therefore no general guidelines could be formulated regarding the choice of antimicrobial treatment under these circumstances. Considering that results of culture and antimicrobial susceptibility testing typically are not available for at least 48 hours after sample collection, it would be rational to repeat bacteriological culture and susceptibility testing at 48 hours intervals on foals hospitalized in neonatal intensive care units, in order to detect on‐going infections or HAIs at an early stage and select effective antimicrobials for treatment. A large proportion of the bacteria isolated after ≥48 hours hospitalization potentially could be the result of HAIs, emphasizing the importance of these infections in foals treated in neonatal intensive care units. Our findings emphasize the need for strategies to prevent and control HAIs in equine hospitals.

## CONFLICT OF INTEREST DECLARATION

Authors declare no conflict of interest.

## OFF‐LABEL ANTIMICROBIAL DECLARATION

Authors declare no off‐label use of antimicrobials.

## INSTITUTIONAL ANIMAL CARE AND USE COMMITTEE (IACUC) OR OTHER APPROVAL DECLARATION

Authors declare no IACUC or other approval was needed.

## HUMAN ETHICS APPROVAL DECLARATION

Authors declare human ethics approval was not needed for this study.

## Supporting information


**Table S1**. MIC Breakpoints used to determine antimicrobial susceptibility of bacteria isolated from foals with sepsis
