# Supplementary material for: Differences in isolation rate and antimicrobial susceptibility of bacteria isolated from foals with sepsis at admission and after ≥48 hours of hospitalization
Source: J Vet Intern Med. 2020 Feb 5;34(2):955–63. doi: 10.1111/jvim.15692 (PMC7096636; doi:10.1111/jvim.15692)
Supplement: Supplementary file 1 — Table S1. MIC Breakpoints used to determine antimicrobial susceptibility of bacteria isolated from foals with sepsis [file JVIM-34-955-s001.pdf]

Supporting Information Table 1. MIC Breakpoints used to determine antimicrobial susceptibility of bacteria isolated from foals with sepsis

Antimicrobial drug	MIC Breakpoint [a]	Special MIC breakpoint (Bacterial species for which this breakpoint was applied)	Bacterial species reported as resistant, regardless of tested MIC [b]
Amikacin	4	-	*Enterococcus* spp.<br>*Streptococcus* spp.<br>*Salmonella* spp.
Ampicillin	8	0.5 (*Actinobacillus* spp.)<br>0.25 (*Streptococcus* spp.)<br>0.25 (*Staphylococcus* spp.)	*Pseudomonas* spp.
Ceftiofur	2	0.25 (*Streptococcus* spp.)	*Enterococcus* spp.
Chloramphenicol	8	-	-
Enrofloxacin	0.5	-	*Enterococcus* spp.
Gentamicin	2	-	*Enterococcus* spp.<br>*Streptococcus* spp.<br>*Salmonella* spp.
Imipenem	1	-	-
Penicillin	0.5	8 (*Enterococcus* spp.)	*Enterobacteriaceae*<br>*Pseudomonas* spp.
Tetracycline	4	2 (*Streptococcus* spp.)	-
Trimethoprim/ Sulfamethoxazole	0.5	-	*Enterococcus* spp.<br>*Streptococcus* spp.<br>*Pseudomonas* spp,

[a] *Standard breakpoint used for all bacterial species (exceptions are specified in the 3rd column)*
[b] *These include bacterial species that are intrinsically resistant to the tested antimicrobial drug or bacterial species for which it is known that in vitro susceptibility results do not correspond well with in vivo efficacy of the drug and are therefore reported as resistant regardless of in vitro test result*

Breakpoints used for Enterobacteriaceae were also applied to Aeromonas spp. and Moraxella spp. isolates.
Breakpoints used for Pseudomonas spp. were also applied to Acinetobacter spp., Ralstonia spp. and Stenotrophomonas spp. isolates and non-enteric isolates that were not further characterized.
Breakpoints used for Streptococcus spp. were also applied to Aerococcus spp. isolates.
Breakpoints used for Staphylococcus spp. were also applied to Arthrobacter spp., Bacillus spp. and Micrococcus spp. isolates.
Breakpoints used for Actinobacillus spp. were also applied to Pasteurella spp. isolates and non-enteric isolates that were not further characterized beyond the level of "non-fermenter."
